# Supplementary material for: FvHsfB1a Gene Improves Thermotolerance in Transgenic Arabidopsis
Source: Plants (Basel). 2025 Aug 2;14(15):2392. doi: 10.3390/plants14152392 (PMC12349616; doi:10.3390/plants14152392)
Supplement: Supplementary file 1 [file plants-14-02392-s001.zip › plants-3753632-supplementary.pdf]

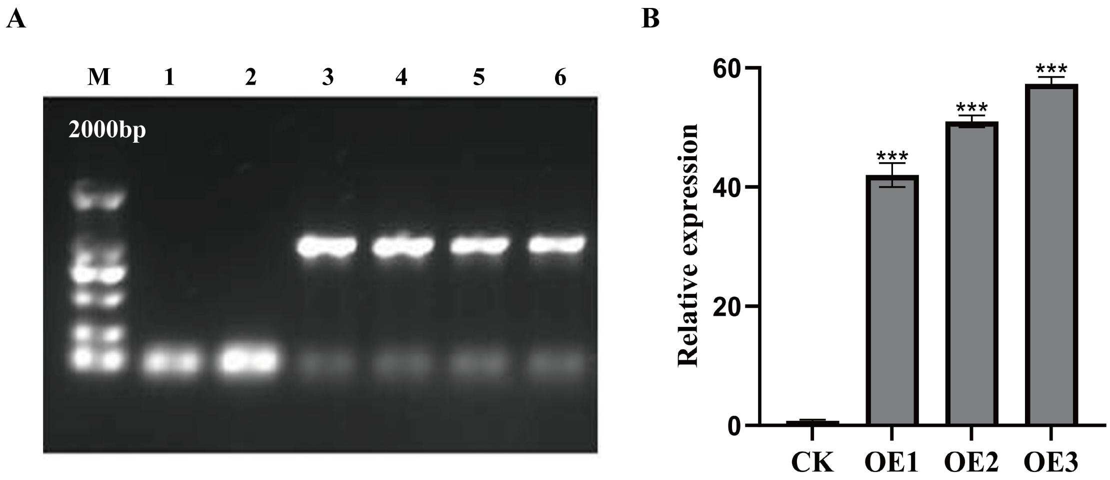

Figure S1: The identification of *FvHsfB1a* transgenic *Arabidopsis thaliana* lines. A: PCR assay of *FvHsfB1a* transgenic lines; B: Relative expression of *FvHsfB1a* gene in different transgenic lines. Note: 1: Wild-type *Arabidopsis thaliana*; 2: Negative; 3: OE line 1; 4: OE line 2; 5: OE line 3; 6: Positive.

Table S1: The primers used in this study.

Gene name	Primer sequence (5′ to 3′)
FvHsfB1a-RT	Forward: CCCATGGTAATGGCGCAAAGGTCAGTT
(XM_004288037.1)	Reverse: GACTAGTTTAGTTGCACACCTTCCCG
FvHsfB1a-GFP	Forward: CCCATGGTAATGGCGCAAAGGTCAGTT
	Reverse: GACTAGTGTTGCACACCTTCCCGCT
FvHsfB1a-qRT	Forward: GTGGAGACGGCGGTGG
	Reverse: TTCAAACCCTCTTCTTCTCCCG
Fvactin-qRT	Forward: ACC GTT GAT TCG CAC AAT TGG TCA TCG
	Reverse: TAC TGC GGG TCG GCA ATC GGA CG
Atactin-qRT	Forward: GAAATCACAGCACTTGCACC
	Reverse: AAGCCTTTGATCTTGAGAGC
AtPOD-qRT	Forward: GACCCTACACTCAACACTAC
(AT1G05250.1)	Reverse: TTCATTGCCTCCACGAATGC
AtSOD-qRT	Forward: TGGCGAAAGGAGTTGCAGTT
(AT2G28190.1)	Reverse: GGCCAGAAACTGTTCCACTCACA
AtP5CS1-qRT	Forward: AGGGAAAGTTCCAGAAAG
(AT2G39800.1)	Reverse: CATAACTAAGCGAGCCAC
AtHSP101-qRT	Forward: ATGTTGGTCACGAGGAAGGT
(AT1G74310.1)	Reverse:TGAAATCGACTGTCCTGCCT
AtHSP70b-qRT	Forward: GAGAGGGCACGAACAAAGGA
(AT5G02490.1)	Reverse: GTCCTCAGCCGACACATTCA
FvWRKY75-AD	Forward: CCCGGGATGGATACCTACCCAACATTCTATT
(XM_004310052.2)	Reverse: CTCGAGTCAGAAAGAAGTGTAGATTTGCATC
FvHsfB1a-PAbAi	Forward: GAGCTCACTTTGGACATGCATGTTAA
	Reverse: CTCGAGAACACAGAGGCTAGAAAATA
pGreenII-SK-	Forward: GAGCTCATGGATACCTACCCAACATTCTATT
FvWRKY75	Reverse:GAATTCTCAGAAAGAAGTGTAGATTTGCATC
pGreenII-0800-	Forward: GTCGACACTTTGGACATGCATGTTAA
FvHsfB1a	Reverse: GGATCCAACACAGAGGCTAGAAAATA
